# The Role of Photobiomodulation and Light Therapy in Enhancing Burn Wound Recovery

**DOI:** 10.1111/jocd.71040

**Published:** 2026-07-28

**Authors:** Edwin Chrabieh, Mahmoud El Hajj, Saif Emsieh, Hazem Omaish, Mazen Kurban

**Affiliations:** ^1^ Faculty of Medicine American University of Beirut Beirut Lebanon; ^2^ Faculty of Medicine University of Jordan Amman Jordan

## Abstract

**Background:**

Photobiomodulation (PBM) is a non‐invasive treatment using low‐intensity red or near‐infrared light to stimulate tissue repair and reduce inflammation. While used in aesthetic and rehabilitative medicine, its role in burn wound healing remains underexplored. Given biological parallels between post‐injury remodeling and cosmetic rejuvenation, a systematic review of PBM in burn care is warranted.

**Aims:**

To evaluate the clinical effectiveness, safety, and outcomes of PBM—such as low‐level laser and light‐emitting diode (LED) therapy—in human burn patients.

**Methods:**

A systematic search of Ovid MEDLINE, Embase, and LILACS was conducted up to 2024. The MEDLINE search was conducted via Ovid MEDLINE, which includes all records indexed in PubMed, ensuring comprehensive coverage of the biomedical literature. Studies were included if they investigated PBM in human burn patients and reported at least one clinical outcome. Two reviewers independently screened studies; a third resolved conflicts. Risk of bias was assessed using a modified Cochrane tool. Due to outcome heterogeneity, narrative synthesis was performed. A binary heatmap visualized patterns across six domains: wound area, healing time, pain (VAS), scar quality (VSS), patient satisfaction, and adverse events.

**Results:**

Seven studies involving 190 patients were included. Healing time and pain (VAS) were most frequently reported, followed by wound area and scar quality. Only one study assessed patient satisfaction, and adverse events were inconsistently reported. PBM showed promising effects, including reduced healing time and pain. However, limited comparable data precluded meta‐analysis.

**Conclusion:**

PBM appears to be a promising adjunct in burn care, with benefits in healing, pain, and scar remodeling. However, methodological limitations, dosing variability, and inconsistent outcome reporting limit conclusions. High‐quality, standardized trials using validated patient‐reported outcomes are needed.

## 1 Introduction and Background

To date, burn injuries remain a complex clinical challenge, frequently resulting in delayed wound healing, persistent inflammation, and disfiguring scarring [1]. Most conventional treatment approaches are limited in their ability to accelerate the healing process or reduce its complications. Thus, there is growing interest in adjunctive therapies that are non‐invasive and able to improve both form and function. Photobiomodulation (PBM) has shown promising results in diverse clinical contexts, but its role in burn wound healing remains underexplored [2]. This review aims to summarize and evaluate the clinical evidence supporting PBM application in burn care and assess whether its effects are significant enough to be incorporated into current treatment strategies.

Photobiomodulation (PBM), also known as low‐level laser therapy (LLLT), refers to the therapeutic use of low‐intensity red or near‐infrared light to stimulate cellular responses and promote tissue healing without generating heat or damage [3]. It was first observed in the 1960s by Endre Mester. In an experiment, Mester noted accelerated hair growth and wound healing in mice exposed to low‐level laser light, and since then, PBM has evolved into a clinically recognized therapeutic approach across multiple domains, including dermatology, rehabilitation, and wound care [4, 5].

PBM works by emitting specific wavelengths of light, usually red light (600–700 nm) or near‐infrared light (700–900 nm), which deliver photons that are absorbed by cellular chromophores such as cytochrome c oxidase (CCO) in the mitochondria, thereby enhancing its activity in the electron transport chain [6]. This stimulation increases the mitochondrial membrane potential and increases the production of adenosine triphosphate (ATP), thus providing the cell with more energy for repair processes [6, 7]. Moreover, a transient increase in reactive oxygen species (ROS) follows. This acts as a secondary messenger to activate transcription factors such as NF‐κB and AP‐1, triggering the expression of genes involved in cell proliferation, anti‐apoptosis, and antioxidant defense [8, 9].

Another known action of PBM is decreasing cellular inflammation [5]. It acts by downregulating pro‐inflammatory cytokines (e.g., TNF‐α, IL‐1β, IL‐6) and promoting anti‐inflammatory signals [10]. Moreover, PBM has been shown to influence immune cell phenotypes, shifting macrophages from a pro‐inflammatory M1 state to a reparative M2 phenotype [11].

PBM is also known to promote angiogenesis [12]. It does this through upregulation of vascular endothelial growth factor (VEGF) and hypoxia‐inducible factor‐1α (HIF‐1α), leading to improved capillary density and blood flow in injured tissues [13, 14]. This increase in blood flow improves oxygen and nutrient delivery, both of which are crucial for healing burned or damaged tissues.

Moreover, PBM has also been shown to stimulate fibroblast proliferation and migration, as well as extracellular matrix (ECM) synthesis, by contributing to an increase in type I collagen [15, 16]. This results in stronger, more organized scar tissue [15, 16]. Studies have shown that PBM accelerates the transition from type III to type I collagen and increases matrix metalloproteinase activity for better tissue remodeling [17]. In addition, PBM is also associated with the activation of latent transforming growth factor beta 1 (TGF‐β1), which is a known regulator of wound healing [18].

The mechanisms enhancing ATP production, angiogenesis, fibroblast activation, and cytokine modulation have been validated in both in vitro and in vivo models [3, 5, 9, 19]. Preclinical studies in animal burn models consistently show faster and improved wound contraction, better epithelialization, and increased collagen production following PBM treatment [20]. For example, a systematic review of 38 animal studies found that PBM significantly enhanced healing outcomes in second‐ and third‐degree burns when used at appropriate wavelengths and energy doses (typically 5–20 J/cm^2^) [20].

Although the biological effects of PBM are well known and established, and the technique is widely used in cosmetic dermatology for wrinkle reduction, its application in burn wound healing has not been systematically explored [21]. Aesthetic uses of PBM rely on similar processes described above: fibroblast stimulation, angiogenesis, and collagen reorganization. These processes are also essential in burn recovery [22]. Furthermore, PBM has shown promising evidence in reducing hypertrophic scars and improving skin texture after burn injuries [23].

Additional animal experiments have strengthened the evidence supporting PBM utility in burn injuries. In rodent burn models, PBM has been shown to elevate TGF‐β1 signaling, increase VEGF expression, and decrease levels of inflammatory cytokines such as TNF‐α, all of which facilitate burn wound recovery [18]. Keratinocyte migration and re‐epithelialization are also enhanced following light therapy, contributing to more rapid wound closure [24]. Mitochondrial assays of cells after PBM confirm increased oxidative phosphorylation in fibroblasts, boosting energy production, which is especially useful after burn injuries when cellular demand is high [6, 7]. In one study, PBM significantly improved collagen alignment and dermal architecture compared to untreated burns, highlighting its role not only in the speed of healing but also in the quality of tissue regeneration [16].

In anti‐aging skin treatments, PBM works by stimulating fibroblasts, enhancing microcirculation, and remodeling collagen [25]. These exact outcomes are also desirable in healing deep dermal wounds. LED‐based PBM is already FDA‐approved for wrinkles and skin rejuvenation, supporting its safe and effective use in humans [22].

Despite these promising findings, there has been no systematic review exploring the role of PBM or light therapy in burn wound recovery. This gap limits the standardized use of PBM after burn injuries, as well as the development of treatment protocols. Given the morbidity, cost, and long‐term disability associated with severe burns, there is a need to explore and validate adjunctive therapies that may be effective and accelerate the healing process.

This review addresses that gap by evaluating current human studies on PBM in burn wound healing. It explores whether PBM offers significant improvements in terms of healing speed, scar quality, and pain. The aim is to translate the established cellular and molecular findings into evidence‐based clinical potential and use.

## 2 Objectives

The primary objective of this systematic review is to critically evaluate the therapeutic impact of photobiomodulation (PBM), including low‐level laser and LED therapies, in enhancing healing outcomes in burn injuries. We examine studies assessing PBM's influence on healing time, scar quality, pain, inflammation, collagen remodeling, and angiogenesis in burn wounds. By combining both clinical and experimental evidence, we aim to determine whether PBM consistently improves these outcomes.

## 3 Innovation

This review is the first to focus exclusively on the role of light therapy in burn wound recovery, distinguishing it from prior reviews that addressed the subject in relation to cosmetic outcomes. It addresses an important need: to provide significant, evidence‐based research on the efficacy of PBM as a potential non‐invasive adjunct therapy for improving healing in burn patients.

Finally, this review highlights how light therapy (PBM), which is already used in anti‐aging and aesthetic medicine, could also help in burn management, as both involve similar tissue repair mechanisms. By showing this connection, we open the door to a potential adjunct therapy for treating wound injuries and improving future clinical care.

## 4 Methods

A comprehensive search of the literature was conducted in three databases: Embase, Ovid MEDLINE, and LILACS. The search strategy included both MeSH terms and keywords related to “photobiomodulation,” “low‐level light therapy,” “LED,” and “burn.” Each database was searched independently. The initial search yielded 2765 records across all databases. After removing duplicates, all remaining titles and abstracts were screened for eligibility.

The screening process was conducted in two phases. Two independent reviewers screened the titles and abstracts, and articles were excluded if they were non‐English, animal or in vitro studies, review articles, conference abstracts, or focused on wounds other than burn injuries or aesthetic procedures unrelated to burns. Full‐text screening was then performed using the same eligibility criteria. Any disagreements between the reviewers were resolved by a third reviewer. In total, 471 full‐text articles were assessed for eligibility, and only 7 met the inclusion criteria and were included in this review.

Eligible studies included randomized controlled trials (RCTs), prospective or retrospective cohort studies, and case series that investigated the use of PBM for the treatment of human burn wounds. Studies had to report at least one of the following clinical outcomes to be included: wound area reduction, healing time, pain (typically assessed by a Visual Analog Scale [26]), scar quality (e.g., Vancouver Scar Scale [VSS]), patient satisfaction, or adverse events.

Data extraction was performed using a standardized spreadsheet. Extracted information included citation and year of publication, study design, patient demographics, burn characteristics (type, location, and depth), intervention details (wavelength, device type, intensity, frequency, and session duration), comparator or control group (if applicable), and all reported outcomes along with the methods used to assess them.

Risk of bias was assessed for each included study. For randomized controlled trials, the Cochrane Risk of Bias 2.0 tool (RoB 2.0) was used, while adapted domains were applied for non‐randomized studies. Each study was evaluated across six standard domains: bias arising from the randomization process, bias due to deviations from intended interventions, bias due to missing outcome data, bias in measurement of the outcome, bias in selection of the reported result, and other potential sources of bias. Two reviewers independently performed the assessments, and conflicts were resolved by a third reviewer.

## 5 Results

### 5.1 Study Selection

A total of 2764 records were identified by searching Embase (*n* = 1678), Ovid MEDLINE (*n* = 877), and LILACS (*n* = 209). After removing duplicates, 2586 articles remained for title and abstract screening. After this stage, 471 articles were selected for full‐text review. Of these, 463 articles were excluded for not meeting the eligibility criteria, leaving 7 studies included in the final systematic review.

**FIGURE 1:**
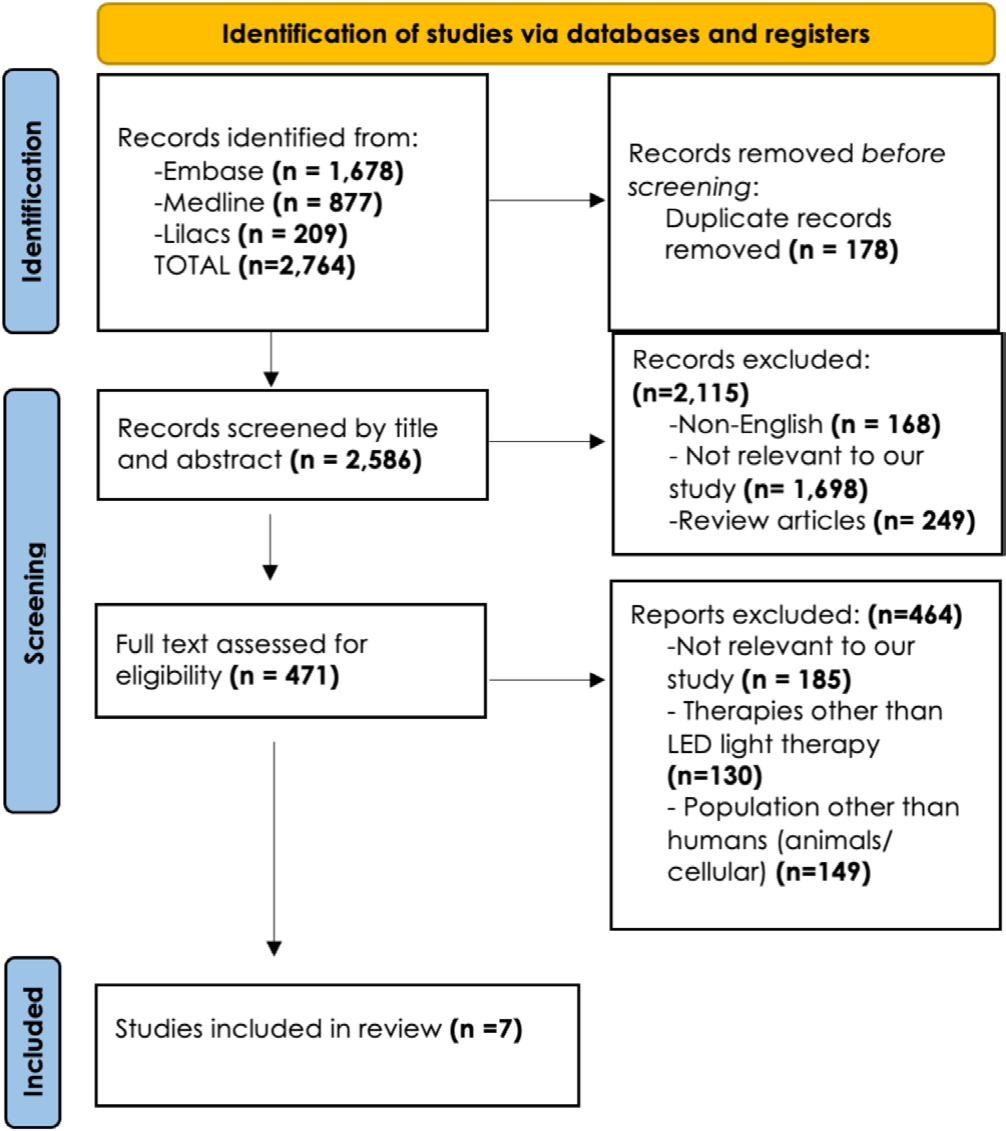
PRISMA flow diagram outlining the selection process for studies included in the systematic review.

A PRISMA flow diagram summarizing the screening process is presented in Figure 1.

### 5.2 Study Characteristics

The seven included studies spanned a publication period from 2004 to 2023 and were conducted across a variety of countries, including Brazil, Italy, China, Germany, and Iran. Study designs were diverse, comprising two randomized controlled trials, one non‐randomized comparative trial, three case series, one intra‐patient controlled study, and one randomized controlled trial protocol. Sample sizes ranged from one patient in case reports and case series to trials with over 60 participants. Most studies enrolled adult patients with partial‐ or full‐thickness burn injuries. Only the study by Kazemikhoo et al. [27] focused exclusively on pediatric patients aged 1 to 14 years. Interventions involved photobiomodulation through low‐level laser therapy (LLLT) and light‐emitting diode (LED) therapy, with wavelengths ranging from 630 to 850 nm. Treatment protocols varied in energy density, session frequency, and duration of follow‐up. All included studies reported on at least one clinical outcome relevant to wound healing.

Methods differed across studies. Vaghardoost et al. [28] used an intra‐patient design in which the donor site was divided into two halves: a control half and a treatment half. This allowed for direct comparison within the same individual. Lu et al. [29] conducted an RCT with defined PBM parameters using LED light at a wavelength of 660 nm and reported mean healing times for both groups. In contrast, de Oliveira et al. [24] and Gaida et al. [30] presented case series. The study conducted by Raffaele et al. [31] included patient satisfaction as a measured outcome. Kazemikhoo et al. [27] compared PBM to traditional split‐thickness skin grafting (STSG) in children and uniquely reported scar incidence outcomes. These examples illustrate the heterogeneity in study design, patient populations, and outcome measurement across the evidence base.

The most frequently reported outcomes were healing time (6 out of 7 studies), pain intensity measured by the visual analog scale (VAS) (6 out of 7), wound area reduction (4 out of 7), and scar quality assessed by the Vancouver Scar Scale (VSS) (2 out of 7). Adverse events were reported in only one study. No studies reported on patient satisfaction. A detailed summary of study characteristics is provided in Table 1.

**TABLE 1:** Characteristics of the included clinical studies evaluating photobiomodulation (PBM) in burn wound healing.

Citation	Country	Study type	Burn type	Device	Wavelength	Sample size	Comparator	Outcome measures	Main findings
Vaghardoost et al. [28]	Iran	RCT (split‐site)	Donor site (Grade 3)	LLLT	810 nm	18	Non‐irradiated side	Wound area, healing time	Significant reduction in wound area on day 7 (*p* = 0.01)
de Oliveira et al. [24]	Brazil	Case series	2nd and 3rd degree	LED	658 nm	5	Self‐control (contralateral limb)	Healing time, pain (VAS)	Faster epithelialization and less pain in irradiated areas
Raffaele et al. [31]	Italy	Case report	Head and neck	LLLT	660 nm	1	None	Healing time	Rapid improvement in edema and wound closure over 5 days
Lu et al. [29]	China	Comparative study	Second‐degree, glucocorticoid‐dependent	LLLT	635–850 nm	62	Standard dressing	Wound area, healing time, pain (VAS)	Faster wound healing and pain reduction with LLLT (*p* < 0.05)
Dahmardehei et al. [32]	Iran	Case series	Type 3 (diabetic patients)	LLLT	650, 810, 660 nm	6 patients (13 ulcers)	None	Wound area, healing time, VSS	Complete graft healing by 8 weeks in all ulcers
Gaida et al. [30]	Germany	Intra‐patient controlled study	Burn scar	LLLT	830 nm	19	Untreated scar area	VSS, pain (VAS)	Improvement in VSS and pruritus (*p* < 0.05)
Kazemikhoo et al. [27]	Iran	Non‐randomized clinical trial	Deep burn ulcers (Grade 2b/3)	LLLT	650 nm	40	STSG	Scar formation, healing time, VSS	89.5% scarless healing with PBM vs. 0% in STSG group

### 5.3 Quantitative Synthesis

A quantitative meta‐analysis (e.g., forest plot) was not performed because of the lack of comparable data across studies. Although several studies reported healing time, only one [29] provided extractable numerical data with means, standard deviations, and a control group comparison. The remaining studies either lacked control groups, used descriptive statistics, or did not report healing time in a format suitable for pooling; therefore, no statistical synthesis could be performed.

### 5.4 Qualitative Synthesis

**FIGURE 2:**
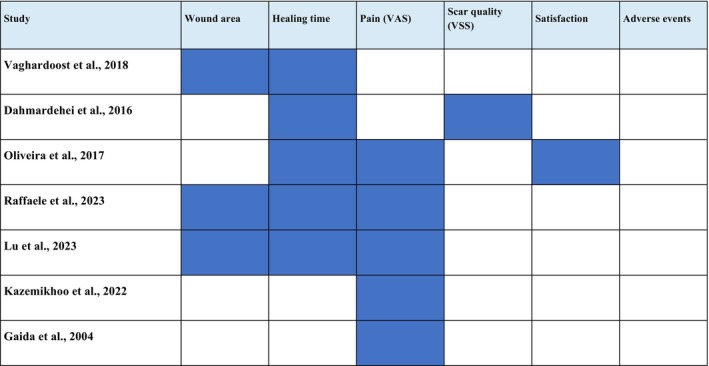
Editable heatmap displaying the presence (blue) or absence (white) of six key clinical outcomes reported across the seven included studies.

Due to heterogeneity in measured outcomes, study design, and treatment protocols, a narrative synthesis was performed. A binary outcome heatmap was developed to facilitate comparison and summarize which outcomes were reported by each study. The reported outcomes were wound area, healing time, pain (VAS), scar quality (VSS), satisfaction, and adverse events. The heatmap is presented in Figure 2 and emphasizes the underreporting of outcomes such as patient satisfaction and adverse events.

### 5.5 Risk of Bias

**FIGURE 3:**
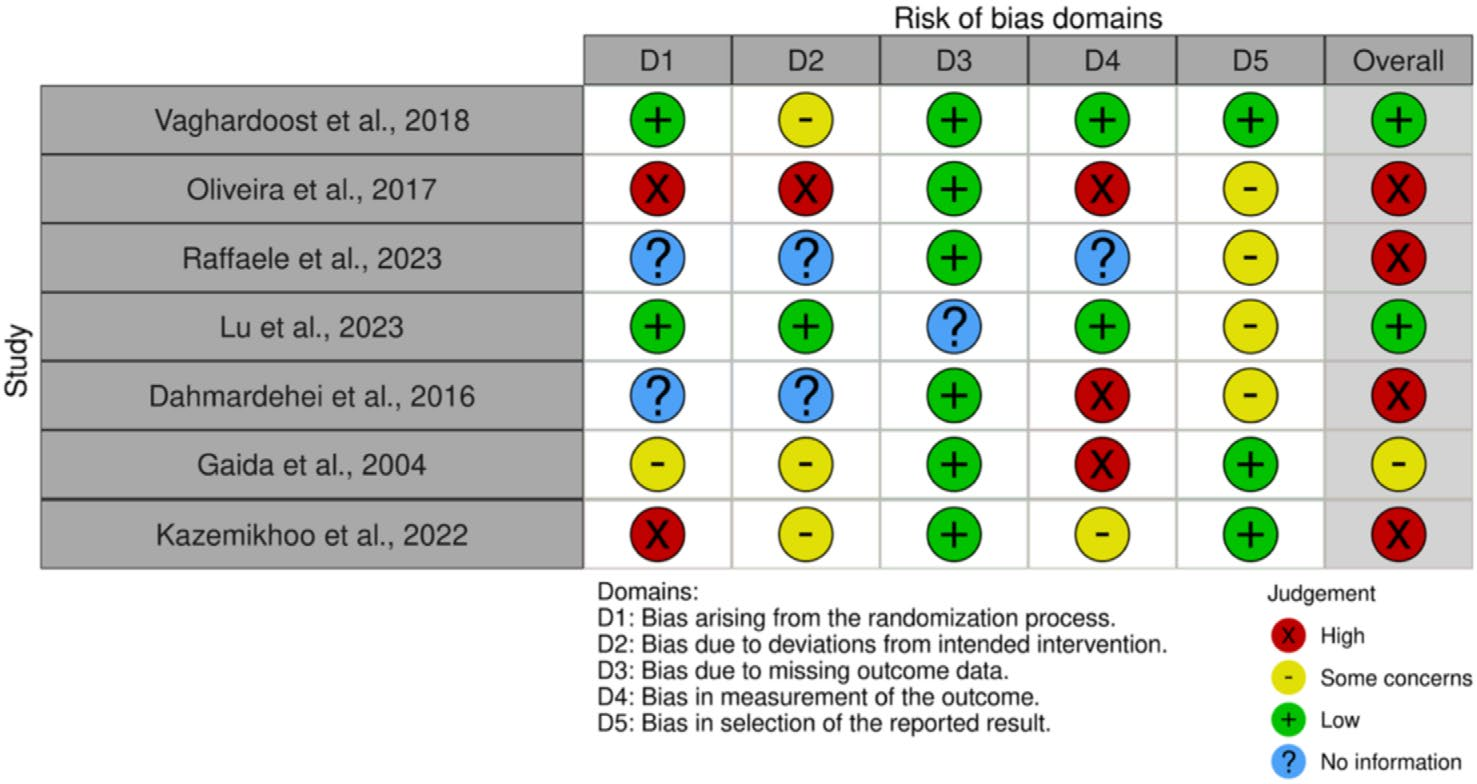
Risk of Bias Summary for Included Studies: A visual summary of the risk of bias assessments across seven included studies using the Cochrane Risk of Bias 2.0 tool (for randomized studies) and an adapted tool (for non‐randomized designs). Domains assessed include randomization, deviations from intended interventions, missing outcome data, measurement of the outcome, selection of the reported result, and other potential sources of bias. Traffic light colors represent low risk (green), some concerns (yellow), and high risk (red).

Of the seven included studies, two were judged to have a low overall risk of bias [28, 29], one had some concerns [30], and four were at high risk of bias [24, 31, 35, 37]. Only the randomized trials clearly described allocation and measurement protocols. The overall distribution of bias levels highlights the need for more regulated and structured studies, such as RCTs, in the future. A visual summary of the risk of bias table is depicted in Figure 3.

## 6 Discussion

Photobiomodulation (PBM), or low‐level light therapy (LLLT), emits specific wavelengths of light that carry photons with energy that, when absorbed by the cell's mitochondria, stimulate cellular processes involved in tissue repair [33]. Because it is non‐invasive, widely considered safe, and easy to use, it has gained considerable interest. Unlike other laser therapies that use high‐energy photons because of shorter wavelengths, LED devices deliver low‐energy light using wavelengths in the red and/or near‐infrared spectrum that penetrate tissue without generating heat, making them well suited for sensitive or damaged skin, such as burned skin.

To further describe PBM's mode of action, photons stimulate cytochrome c oxidase in the mitochondria. This increases adenosine triphosphate (ATP) production in the electron transport chain (ETC), modulates reactive oxygen species, and triggers intracellular signaling pathways associated with cellular proliferation, migration, and angiogenesis [5, 6, 34]. These effects are especially important for accelerating wound healing and regeneration. In the context of burn injuries, light therapy has been associated with faster re‐epithelialization, enhanced fibroblast activity, and a reduced inflammatory response [35].

Despite its promising mode of action in situations that require enhanced wound healing, such as burn injuries, clinical research on this subject is scarce, and PBM therefore remains underutilized in burn care protocols. We focused on domains such as healing time, pain, scar formation, patient satisfaction, and adverse events. This review is subject to certain limitations. Only English‐language studies were included, which may introduce language bias and result in the exclusion of relevant studies published in other languages.

### 6.1 Accelerated Healing Time

Healing time was one of the most reported outcomes among the included studies. In almost all of the articles, PBM‐treated groups demonstrated faster re‐epithelialization and wound closure compared to controls. For example, the study conducted by Lu et al. [29] reported a mean reduction of 4.7 days in healing time for superficial partial‐thickness burns treated with LED therapy, while Raffaele et al. [31] observed a 5‐day improvement following low‐level laser therapy. Although comparison of these results is difficult because of differences in study design and reporting standards, the direction of effect was consistently toward PBM accelerating burn wound recovery.

These clinical observations are consistent with animal studies. A systematic review conducted by Deana et al. [20] analyzing 38 animal studies found that PBM significantly enhanced wound contraction and epithelialization in burn models. Histological analysis showed earlier granulation tissue formation, reduced inflammation, and increased neovascularization, all of which point toward tissue regeneration. These effects are believed to be due to the mode of action of PBM in increasing ATP production, which allows enhanced cellular proliferation.

However, unlike animal models in which PBM parameters were well controlled, clinical studies varied in their intervention protocols. There was no consensus to follow in any of the above studies, and they differed in wavelength, energy density, session frequency, and treatment duration. This limited the ability to generalize findings. To better address this issue, future trials should use standardized PBM protocols and clearly report treatment parameters.

### 6.2 Pain Reduction and Inflammatory Modulation

Pain is a significant factor when dealing with burn patients as it extends beyond the acute phase and negatively affects the patient's recovery and quality of life. Five of the seven included studies assessed pain using the Visual Analog Scale (VAS), with all reporting reductions in pain levels following PBM treatment. Gaida et al. [30] documented a 57% reduction in VAS scores after five PBM sessions, while de Oliveira et al. [24] observed progressive pain relief during the first week of LED therapy.

These analgesic effects are supported by the fact that PBM can suppress pro‐inflammatory cytokines such as tumor necrosis factor‐alpha (TNF‐α) and interleukin‐6 (IL‐6), reduce oxidative stress in nociceptive neurons, and downregulate nerve growth factor (NGF) expression [14, 36]. Moreover, modulation of transient receptor potential (TRP) channels and enhancement of local microcirculation can also contribute to pain reduction.

It is important to note that the analgesic benefits of PBM were demonstrated for many conditions other than burn injuries. Light therapy showed efficacy in treating oral mucositis, diabetic neuropathy, and musculoskeletal pain, further highlighting its neuromodulatory and anti‐inflammatory capabilities [36, 37]. PBM in the context of burn care offers a useful adjunct to pharmacologic therapy in order to minimize opioid dependence.

However, the lack of consensus in treatment protocols and pain assessment methods across studies remains a barrier to application in clinical settings. More trials should use well‐regulated PBM exposure doses, validated pain scales, and consistent outcome reporting to strengthen the evidence base.

### 6.3 Scar Remodeling and Aesthetic Outcomes

The formation of scars remains a major concern after burn injuries because of its impact on appearance and function. Three studies in this review assessed scar quality using the Vancouver Scar Scale (VSS), which evaluates vascularity, pigmentation, pliability, and height. Dahmardehei et al. [32] reported significantly better VSS scores in the PBM‐treated group, especially in the domains of pigmentation and vascularity. de Oliveira et al. [24] described qualitative improvements in scar pliability and coloration, although outcomes were not scored quantitatively.

These clinical observations align with the known effects of PBM on tissue remodeling. PBM has been shown to modulate transforming growth factor‐beta 1 (TGF‐β1), regulate the balance between type I and type III collagen, and upregulate matrix metalloproteinases, all of which contribute to more organized extracellular matrix deposition and improved scar architecture [8, 9]. These mechanisms play an important role in scar formation and have been shown in both laboratory and clinical models.

### 6.4 Wound Area Reduction

Wound area reduction was another relevant parameter reported in several studies. Vaghardoost et al. conducted an RCT assessing the impact of PBM on donor site healing after split‐thickness skin grafting in burn patients. The results showed a statistically significant reduction in wound size in the treated group compared to the control group by day 7 (6.6 ± 3.13 cm^2^ vs. 13.7 ± 6.71 cm^2^, *p* = 0.01), confirming the efficacy of PBM in accelerating wound closure [28]. Similarly, de Oliveira et al. observed faster reduction in wound area in patients with bilateral burns treated with LED therapy, though quantitative values were not explicitly reported [24]. Raffaele et al., although a case report, described a decrease in visible wound size over five sessions of low‐level laser therapy, with clear visual improvement observed as early as 72 h into treatment [31]. These findings further show that PBM contributes meaningfully to wound contraction and recovery. However, the limited number of studies reporting this outcome quantitatively highlights the need for standardized wound assessment methods in the future.

### 6.5 Supplemental Analysis of Additional Outcomes

In addition to the primary outcomes of healing time, pain, and scar quality, many studies also reported secondary outcomes. Kazemikhoo et al. [27] documented a reduced incidence of wound infections in the PBM‐treated group compared to those receiving split‐thickness skin grafting (STSG), suggesting a potential role for PBM in decreasing infection rates in burn injuries.

Another important symptom in burn injuries that is often overlooked is pruritus. This outcome was addressed by two studies. Gaida et al. [30] and de Oliveira et al. [24] reported reductions in itch intensity among PBM‐treated patients, likely due to the therapy's anti‐inflammatory effects. Since pruritus can interfere with sleep, emotional well‐being, and wound healing rate, its inclusion as a primary outcome in future trials is recommended.

### 6.6 Safety, Satisfaction, and Clinical Feasibility

All included studies consistently reported PBM as a safe and well‐tolerated therapy. No trials documented adverse events attributable to the intervention. This further highlights that this type of therapy has a very low risk when administered within therapeutic parameters. This is especially important in the context of burn care, where skin barrier compromise increases susceptibility to infection and other complications, and the absence of adverse effects is reassuring.

PBM is also practical and easy to integrate clinically. It can be delivered using portable or stationary LED or laser devices, requires minimal training, and is suitable for both inpatient and outpatient settings. The fact that it does not directly contact the skin and is a painless, short therapy makes it appropriate for patients with hypersensitive or painful wounds. Despite all of these advantages, it remains unclear whether PBM has significant clinical effects on burn injuries in humans because of the lack of research studies. Only one study (Raffaele et al.) assessed patient satisfaction, noting that 86% of patients treated with PBM expressed high satisfaction, compared to 58% in the control group [31].

### 6.7 Evidence Gaps and Methodological Challenges

Although the reviewed studies all favor PBM's role as an adjunct therapy in burn injury treatment, the quality and consistency of the clinical evidence are limited. Most trials had small sample sizes, lacked randomization, and reported incomplete or inconsistent outcome measures. Significant heterogeneity in treatment parameters, such as wavelength (ranging from 630 to 980 nm), energy density (1–10 J/cm^2^), frequency of sessions, and duration, differed across studies. The variation in methods also hindered the ability to conduct a meta‐analysis. While a forest plot was initially considered, only one study provided sufficient data for comparison. Inadequate documentation of control conditions, patient characteristics, and measurement protocols further reduced comparability.

Addressing these issues will be crucial for establishing a solid evidence base capable of standardizing clinical guidelines and optimizing protocols.

## 7 Impact of the Work

This systematic review is the first to focus on how photobiomodulation (PBM) can help with burn wound healing in human patients. By combining results from different types of clinical studies and patient groups, it shows that PBM is consistently linked to faster healing, less pain, better scar quality, and strong safety. These results are supported by PBM's well‐known mechanism of action, including its ability to improve cellular energy production, reduce inflammation, support new blood vessel growth, and help the skin rebuild itself. The review also examines evidence from animal studies and cosmetic treatments to suggest ways to improve research quality and support the idea that PBM can be safely and effectively used in burn care.

## 8 Future Directions

To help PBM become a regular part of burn treatment, future studies need to be carefully planned. Researchers should conduct high‐quality randomized controlled trials with more participants, longer follow‐up times, and clear treatment details. It is important to use consistent settings, such as light wavelength, energy level, frequency of use, and session duration, so results can be compared across studies. These trials should measure both medical outcomes (such as healing time, scar improvement, and infection rates) and patient‐reported outcomes, using trusted tools such as pain scales and quality‐of‐life questionnaires. Researchers should also study how PBM works on different types of burns, body areas, and populations with special needs, such as children or those with other health issues. Lessons from animal studies and cosmetic medicine research could also help improve how PBM is used in burn care.

In conclusion, PBM appears to be a promising adjunctive treatment in burn wound care, with potential benefits in healing time, pain management, and scar remodeling. However, limitations in study design, variability in dosing protocols, and inconsistent outcome reporting limit definitive conclusions. Additionally, heterogeneity in burn characteristics, initial management, and practitioner expertise may further influence outcomes, highlighting the absence of a standardized treatment approach. High‐quality, standardized trials with validated patient‐reported outcomes are needed to establish optimal protocols and confirm clinical efficacy.

## Ethics Statement

The authors have nothing to report.

## Consent

Inapplicable systematic review.

## Conflicts of Interest

The authors declare no conflicts of interest.

## Data Availability

The data that support the findings of this study are openly available in PubMed Central (PMC) at https://www.ncbi.nlm.nih.gov/pmc/, reference number PMID.
